# Neuronal mechanisms of dynamic strategic competition

**DOI:** 10.21203/rs.3.rs-2524549/v1

**Published:** 2023-03-20

**Authors:** Yaoguang Jiang, Kelsey R. McDonald, John M. Pearson, Michael L. Platt

**Affiliations:** 1. Department of Neuroscience, Perelman School of Medicine, University of Pennsylvania, Philadelphia, PA, 19104, USA; 2. Department of Psychology, School of Arts and Sciences, University of Pennsylvania, Philadelphia, PA, 19104, USA; 3. Marketing Department, the Wharton School, University of Pennsylvania, Philadelphia, PA, 19104, USA; 4. Center for Cognitive Neuroscience, Duke University, Durham, NC, 27708, USA; 5. Department of Psychology and Neuroscience, Duke University, Durham, NC, 27708, USA; 6. Department of Biostatistics and Bioinformatics, Duke University, Durham, NC, 27708, USA

## Abstract

Competitive social interactions, as in chess or poker, often involve multiple moves and countermoves deployed tactically within a broader strategic plan. Such maneuvers are supported by mentalizing or theory-of-mind—reasoning about the beliefs, plans, and goals of an opponent. The neuronal mechanisms underlying strategic competition remain largely unknown. To address this gap, we studied humans and monkeys playing a virtual soccer game featuring continuous competitive interactions. Humans and monkeys deployed similar tactics within broadly identical strategies, which featured unpredictable trajectories and precise timing for kickers, and responsiveness to opponents for goalies. We used Gaussian Process (GP) classification to decompose continuous gameplay into a series of discrete decisions predicated on the evolving states of self and opponent. We extracted relevant model parameters as regressors for neuronal activity in macaque mid-superior temporal sulcus (mSTS), the putative homolog of human temporo-parietal junction (TPJ), an area selectively engaged during strategic social interactions. We discovered two spatially-segregated populations of mSTS neurons that signaled actions of self and opponent, sensitivities to state changes, and previous and current trial outcomes. Inactivating mSTS reduced kicker unpredictability and impaired goalie responsiveness. These findings demonstrate mSTS neurons multiplex information about the current states of self and opponent as well as history of previous interactions to support ongoing strategic competition, consistent with hemodynamic activity found in human TPJ.

## Introduction

Competitive interactions occur in an evolving decision space dynamically reshaped by one’s own behavior and the behavior of others (Platt & Ghazanfar, 2004; Platt et al., 2016). In *The Art of War*, the Chinese military theoretician Sun Tzu distinguished between strategy and tactics in the context of iterated competitive interactions (Tzu, 2008). Strategy is the plan or set of goals one hopes to achieve, whereas tactics are the specific actions or steps enacted to accomplish that strategy. Strategies are long-term and slow to adjust while tactics are short-term and dynamic. In war, sports, or business, both strategy and tactics are necessary: “Strategy without tactics is the slowest route to victory. Tactics without strategy is the noise before the defeat” (Tzu, 2008). Deft deployment of tactical maneuvers to achieve strategic goals, such as acquiring resources or mating opportunities through aggression, affiliation, or cooperation, has been theorized as a major selective force shaping the evolution of sophisticated cognition and concomitant increases in brain size in the primate clade—the so-called “Machiavellian Intelligence Hypothesis” (Whiten & Byrne, 1988).

Although real-life social interactions are undeniably complex, for simplification most studies in social neuroscience have employed tasks featuring discrete, static, typically binary options, such as whether to sacrifice or save another person (Thomson, 1976; Cikara et al., 2010), to betray or cooperate with a partner (Rilling et al., 2002; Stephens et al., 2002), or how much to donate to or invest in someone (Güth et al., 1982; Forsythe et al., 1994; Sanfey et al., 2003; Chang et al., 2012). This approach has uncovered key nodes in these ‘social brain network’ (Lee, 2008; Rushworth et al., 2013; Lee & Seo, 2016; Tremblay et al., 2017; Wittmann et al., 2018), but it often fails to capture the complexity, dynamics, and state-dependency of real-life interactions (Scholl et al., 2015; Camerer & Mobbs, 2017), and leaves the distinction between tactics and strategy unaddressed (Hillman & Bilkey, 2012; Spielberg et al., 2013).

Many forms of complex human social behaviors, such as altruism (Morishima et al., 2012), cooperation (Lissek et al., 2008), deception (Bhatt et al., 2010; Carter et al., 2012), and moral judgment (Gallagher et al., 2000; Saxe et al., 2004; Brüne & Brüne-Cohrs, 2006; Young et al., 2007), rely on constructing and maintaining rich models of the beliefs, desires, goals, and tendencies of others. Within the human social brain network, the temporo-parietal junction (TPJ) has been implicated in these computational processes, often referred to as mentalizing or theory-of-mind (ToM) (Carter & Huettel, 2013). For example, hemodynamic activity in TPJ tracks strategic deception in bargaining (Bhatt et al., 2010), and differentiates between human and computer opponents in poker (Carter et al., 2012). These observations, and others, provoke the hypothesis that TPJ computes models of other agents whose tactics and strategies can influence one’s own behavior (Saxe, 2006; Carter & Huettel, 2013). The mechanisms underlying these hemodynamic signals in TPJ, however, remain poorly understood, in part due to challenges probing dynamic, strategic social interactions in nonhuman animals in which neuronal activity is more readily studied directly.

fMRI-based resting state connectivity studies identified primate middle superior temporal sulcus (mSTS) as the potential homolog of human TPJ (Mars et al., 2013; Roumazeilles et al., 2021). mSTS neurons in macaques respond to social perpetual stimuli such as faces, bodies, biological motion, and eye gaze (Perrett et al., 1985; Puce & Perrett, 2003; Tsao et al., 2003, 2008; Popivanov et al., 2012). Further, BOLD signals within face patches in macaque STS, including mSTS, are selectively enhanced by observing social interactions between other monkeys compared with interactions among objects (Sliwa & Freiwald, 2017). Building upon these observations, Ong and colleagues (2021) found mSTS neurons encode a rich array of abstract information supporting cooperation, including goals and strategic tendencies of partners, their deviations from predicted strategies, and history of reward outcomes. Importantly, these signals are not reducible to perceptual cues, but instead reflect the sophistication of the computational model that best accounts for the behavior of the partner. These findings lend initial support to the hypothesis that the macaque mSTS is involved in the process of computing and updating the goals, intentions, tactics, and strategies of others during social interactions, consistent with functions of human TPJ.

Despite these observations, open questions remain. First, the macaque mSTS consists of multiple functional subregions along the upper and lower banks of the superior temporal sulcus (Tsao et al., 2003, 2008; Sliwa & Freiwald, 2017) and their precise correspondence to human TPJ are unclear (Mars et al. 2013; Ong et al., 2021; Roumazeilles et al., 2021). Second, whether mSTS neurons dynamically encode information supporting tactical maneuvers during strategic competition remains unknown. Third, the functional contributions of mSTS to strategic social interactions have not been tested through causal manipulations.

To address these questions, we compared the behaviors of humans and rhesus macaques (*Macaca mulatta*) playing a virtual two-player competitive soccer game (Penalty Kick, hereafter PK), based on the classic zero-sum payout structure of the “matching pennies” game (Durlauf & Blume, 2010; Camerer, 2011). The PK game captures the richness of real-life competition (Camerer & Mobbs, 2017) by creating an action space continuously reshaped by the behaviors of both players. Within this space, each player’s tactical maneuvers can be quantified, and their strategies can be disentangled from the strategies of their opponent. We found that humans and monkeys deployed similar tactics within virtually identical strategies, suggesting strategic competition is supported by common underlying mechanisms in both species.

Our group recently reported increased BOLD signals in TPJ and dorsolateral prefrontal cortex (dlPFC) in humans playing PK against a human opponent compared with playing against a computer, and these signals were correlated with individual sensitivity to one’s own strategy as well as the strategy of the opponent (MacDonal et al., 2020). We hypothesized that if mSTS subserves functions similar to those performed by human TPJ, as would be predicted if these areas are homologous, then neurons in mSTS should represent information used to select tactical maneuvers and update strategies. Further, pharmacological inactivation of mSTS should degrade performance by impairing the deployment of tactics and the maintenance of strategies.

Here we report evidence supporting all these predictions. We uncovered two spatially segregated populations of neurons with different tonic firing rates and diametric activity patterns during gameplay. Firing rates of both populations carried information about the actions and states of self and opponent, reward outcomes, social context, and the history of interactions within the game. Pharmacological inactivation of mSTS reduced kicker unpredictability and impaired goalie responsiveness, thereby downgrading strategic complexity. Together, our findings provide further physiological and functional support for the hypothesis that mSTS carries out core functional computations performed by human TPJ during strategic social interactions.

## Results

### Experimental paradigm

**Figure 1.**
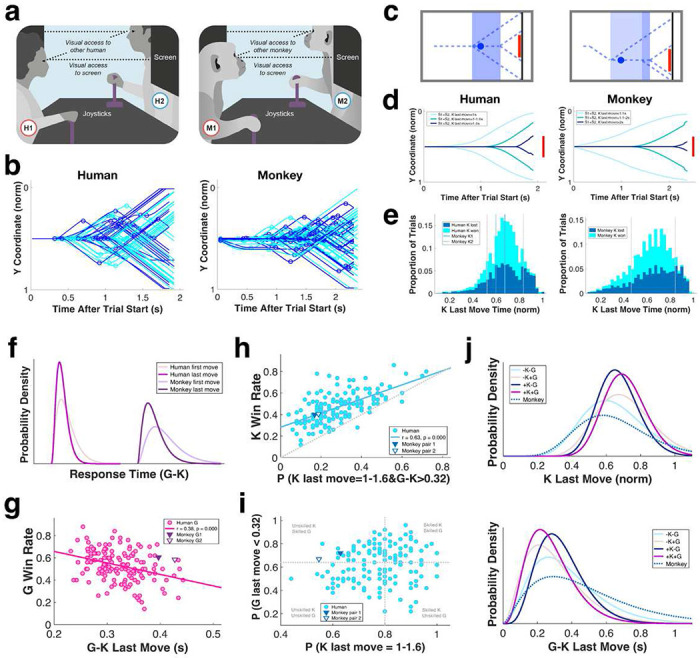
Humans and monkeys play a virtual soccer in identical setups and demonstrate comparable grasp of the rules and dynamics of the game. **a:** Experimental setup. Pairs of human or monkey players sat across from (and with full visual access to) each other, each facing their own screen and using their own joystick to manipulate ball/bar movement. **b:** Movement trajectories of human (**left**) and monkey (**right**) kicker in 50 consecutive trials. X axis, time. Y axis, normalized vertical axis on screen. Circles mark ball direction change, defined as sudden (<0.06 s) changes in ball direction. Blue lines, kicker losing trials; cyan lines, kicker winning trials. **c:** The theoretical advantageous time window (shaded in blue) for kicker’s last move when 1) both kicker and goalie are centered (**left**), and 2) both kicker and goalie are displaced from the center of the screen (**right**) at the moment of kicker’s last move. **d:** Average ball trajectories for all S1 (straight up) and S2 (straight down) trials in which human (**left**) and monkey (**right**) kickers timed the last move 1) before, 2) within, and 3) after the advantageous window. Straight red line represents the goalie bar. X axis, time. Y axis, normalized vertical axis on screen. **e:** Distributions of time of last movement for all human (**left**) and monkey (**right**) kickers’ winning and losing trials. Dotted gray lines mark the theoretical advantageous time window for kicker last move. Average last move times for monkey kickers are superimposed for comparison. **f:** Gamma-fitted distributions of goalies’ reaction times to the first and last movements of human and monkey kickers. **g:** Overall, slow reaction to kicker’s last move negatively impacts goalie performance. Each datapoint represents one human subject; average reaction time and win rate for monkey goalies are superimposed for comparison. Straight line, linear fit. **h:** Together, kicker last move timing (whether it is within the advantageous window) and goalie last move RT (whether it is faster than average) can reliably predict game outcome. Straight line, linear fit. **i:** Kicker and goalie last move timings are independent dimensions that can be used to untangle kicker and goalie competencies. Each datapoint represents one human subject; average timing and win rate for monkeys are superimposed for comparison. **j:** Gamma-fitted distributions of kickers’ last move time (**top**) and goalies’ RTs (**bottom**) for all human kicker-goalie competency combinations. Corresponding distributions for monkeys are superimposed for comparison.

We first studied the behaviors of human and monkey ‘kickers’ playing penalty kick (hereafter PK) against conspecific ‘goalies’ in virtually identical setups (Figures 1a–b). Each participant faced a monitor and had full visual access to their opponent. A trial started with a blue disc (the ‘ball’) traveling from left to right across the screen at a constant speed. Kickers used a joystick to move the ball up and down, and goalies used a joystick to move a red rectangle (the ‘goalie bar’) up and down to block the ball. A trial ended when either the kicker successfully maneuvered the ball to the ‘goal line’ located behind the goalie bar, or the goalie intercepted the ball with the bar (see also Methods and Supplementary Videos 1-3).

### Humans and monkeys played the game very similarly

PK is strictly competitive, like chess, and its payoff structure is the same as the classic matching pennies game (Camerer, 1997, 2011). The objective of the goalie is always to match, whereas the objective of the kicker is always to mismatch, the opponent. Because interactions between kickers and goalies in PK are dynamic and extend in real time, reaction time (RT) is also critical to performance. PK can thus be divided into an initial game of movement sequences, like chess, and a later game of timing, like dueling (8, 9, Supplementary Figures 1a-e). In dueling, one tries to find the most advantageous time to fire the bullet, which reflects the competing risk of firing too early and missing the opponent or firing too late and being shot. Similarly, in PK, there is a strategically advantageous time window, determined by the speeds of the ball and the goalie bar, on the one hand, and visuo-motor reaction times, on the other, for the kicker to initiate the final ball movement. Acting too early, the kicker leaves the goalie too much time to react but acting too late reduces the likelihood of successfully bypassing the goalie (Figures 1c–d, Supplementary Figure 1f, see also Methods). Extensive behavioral data (n = 151 pairs of humans playing a total of 7550 trials, 2 pairs of monkeys playing a total of 11400 trials) revealed humans and monkeys playing as kickers effectively timed their last moves (humans, 78.0%; monkeys, 60.2%; Figure 1e) within this strategically advantageous window (humans, 1-1.6s; monkeys, 1.1-2s), despite minor imprecision in tactical implementation from trial to trial (for example see Supplementary Figure 1g). These observations indicate similar grasp of the temporal dynamics of the game by both monkeys and humans.

Goalie reaction time to the kicker’s last movement also strongly impacts game outcome. Both human and monkey goalies sharply reacted to kickers’ final moves, but not other moves (median RT = 0.32s for humans, 0.40s for monkeys, Figure 1f). Faster RTs corresponded to higher win rates for goalies (Figure 1g). On those trials where the goalie did not move in response to the kicker’s last move (i.e., last move ‘RT’ < 0), they were more likely to win, suggesting the goalie correctly anticipated the kicker’s last move rather than failing to respond to it (Supplementary Figure 1h). Again, monkeys and humans deployed similar tactics as goalies, suggesting consistency in their grasp of the dynamics of the game.

We found the timing of kicker and goalie final moves alone predicted the winner on ~70% of trials (human: 71%, monkey: 67%, Figure 1h). For humans, kicker and goalie last move reaction times were uncorrelated (r = 0.13, P = 0.121) and thus could be used to disentangle the independent performance of kicker and goalie within any pair of players (Figure 1i). Using this approach, all 151 human pairs could be classified into unskilled kicker-unskilled goalie (−K−G), unskilled kicker-skilled goalie (−K+G), skilled kicker-unskilled goalie (+K−G), and skilled kicker-skilled goalie (+K+G) pairs based on the timing precision of their last movements, regardless of trial outcome (Figure 1i, Supplementary Figure 1i). Playing against a skilled goalie improved a kicker’s last move timing, and, likewise, playing against a skilled kicker improved a goalie’s final move reaction time (Figure 1j). We also found skilled kickers improved their last move timing as they played more trials (Supplementary Figure 1j), further emphasizing the dynamic, interactive, evolving nature of the game. Importantly, not only did movement trajectories deployed by monkeys resemble those used by humans (for examples see Figures 1b, d), but reaction times of monkey players also fell within the range of variation of human players (for example see Figures 1e, g, i). Monkeys demonstrated a command and execution of virtual soccer on par with an unskilled human kicker and a moderately skilled human goalie (Figure 1i).

### Strategic components of performance

**Figure 2.**
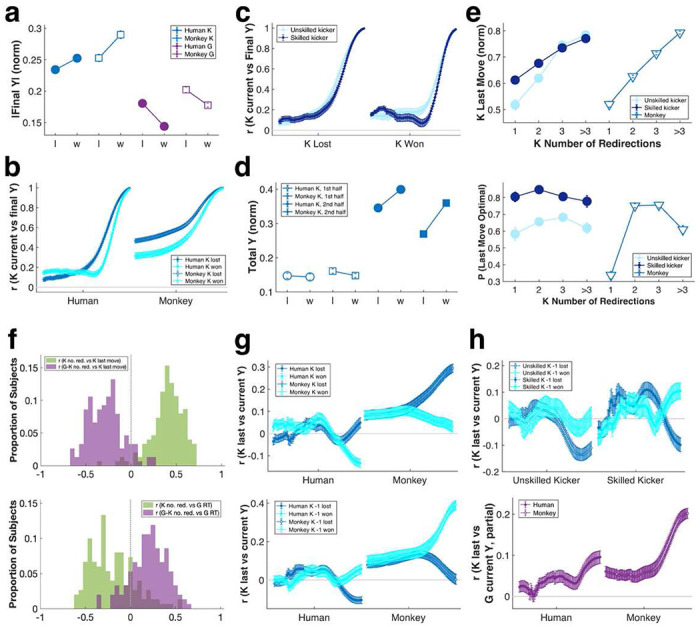
Humans and monkeys play virtual soccer using similar strategies and tactics. **a:** In winning trials, kickers moved the ball further away, and correspondingly displaced goalies more. **b:** Unpredictability in this final move, measured as lower overall correlations between mid-trial and final ball positions, further improved kickers’ chance of winning. **c:** Skillful kickers were exceptionally good in thus obscuring their final intentions. **d:** The successful tactic was to minimize and randomize ball movements in the earlier part of the trial and make large movements only in the later part of the trial. **e:** Total number of kicker moves impacted last move timing (**top**) and its success (**bottom**). **f:** Total number of kicker moves and the relative number of goalie minus kicker moves impacted kickers’ last move timing (**top**) as well as goalies’ RT to last move (**bottom**). **g: Top:** In winning trials, kickers’ movements less resembled those from the previous trials. **Bottom:** Winning a trial led kickers to repeat previous trajectories. **h: Top:** Both unskilled and skilled kickers deployed win-stay, lose-shift strategies. **Bottom:** For unskililed kickers this tendency was successfully exploited by goalies. X axis, time. Error bars, mean ± SEM.

Next, we explored other tactical components beyond movement timing. Both human and monkey kickers were more likely to score by displacing the ball further from its starting position (normalized ball displacement, from 0 = not moving from midline to 0.5 = at the top or bottom edge of the screen: human kicker losses = 0.234 ± 0.004 and wins = 0.252 ± 0.004; monkey kicker losses = 0.253 ± 0.006 and wins = 0.290 ± 0.006). Understandably, larger ball movements elicited greater displacements of the goalie bar (Figure 2a). Ball displacement was most effective when it was unpredictable, as shown by overall lower correlations between mid-trial and end-of-trial ball positions in trials kickers won (Figure 2b). In other words, kickers improved their chance of winning by obscuring the direction of their final movement. Humans were overall better than monkeys (Figure 2b), and skilled human kickers were especially proficient at this tactic (Figure 2c). Although total ball movement was positively correlated with the likelihood of kicker winning (Supplementary Figure 2a), it was only in the latter half of the trial that most ball displacement occurred, and the magnitude of this terminal displacement most significantly impacted winning (ball displacement, human, first half: kicker losing trials = 0.147 ± 0.008, winning trials = 0.144 ± 0.008; human, second half: kicker losing trials = 0.346 ± 0.004, winning trials = 0.400 ± 0.004; monkey, first half: kicker losing trials = 0.161 ± 0.006, winning trials = 0.148 ± 0.006; monkey, second half: kicker losing trials = 0.269 ± 0.006, winning trials = 0.360 ± 0.005) (Figure 2d).

Although ball movement preceding the final shot did not directly impact a kicker’s chance of winning, most kickers made multiple movements on each trial (number of moves/trial, human kicker, mean = 2.52 ± 0.07, range = 0-16; monkey kicker, mean = 2.25 ± 0.08, range = 0-19). Compared with straight shots, making multiple moves significantly improved both monkey and human kicker’s chances of winning (Supplementary Figure 2b), in part because the total number of movements interacted significantly with the timing of the last shot. Specifically, the more movements a kicker made, the later in time they executed their final move. As the total number of moves increased, the latency for last move increased most steeply for monkey kickers and least steeply for skilled human kickers (Figure 2e, top). Nevertheless, for all kickers, making multiple moves improved the likelihood of executing the final move within the advantageous time window, but extended staggering of movements imposed an additional risk of making a final shot too late to evade the goalie bar (Figure 2e, bottom).

Goalies also made multiple movements during gameplay to closely follow changes in ball position, and this tactic often resulted in kickers making their final shot too soon (Figure 1f, top; see also Supplementary Figure 2c). Thus, multiple moves by the kicker improved the timing of the kicker’s final shot, while multiple moves by the goalie in response impaired it. Yoked temporal dynamics of kickers and goalies were also evident in the fact that, as goalies increased the number of movements they made, they also increased their RT to kickers’ final move. By contrast, when kickers increased their total number of movements this lowered goalie RT (Figure 1f, bottom). In other words, when kickers made multiple moves this may have exposed the intended direction of their final shot to the goalie, but when goalies closely matched movements of the ball this appeared to impair their ability to react swiftly to the kicker’s final shot.

Kicker strategy thus consisted of initially making several small movements followed by a sudden, large displacement of the ball near the end of the trial. Goalie strategy reflected the tradeoff of responding to every move made by the kicker and preparing to respond to the final shot. Notably, the deployment of tactics by the same player differed greatly from one trial to the next (for example see again Figure 1b). This trial-to-trial variability was also key to kicker performance. Specifically, the less the current pattern of movements resembled those on the preceding trial, the more likely it was for the kicker to score (Figure 2g, top). Despite this clear strategic advantage in maintaining trial-to-trial unpredictability, however, all players, including the most skilled, often relied on a simple ‘win-stay, lose-shift’ strategy (Figure 2g, bottom, Figure 2h, top, Supplementary Figure 2d). This tendency for kickers to repeat successful tactics from the last trial was exploited by goalies, evident in the partial correlation between the trajectory of the kicker on the preceding trial and the trajectory of the goalie on the current trial (Figure 2h, bottom, Supplementary Figure 2e). Nevertheless, goalies did not gain additional advantage by anticipating win-stay tendencies when playing against skilled kickers, whose final shots were sharp and precisely-timed (Supplementary Figure 2f). Monkeys deployed similar tactics and exhibited similar strategic tendencies as human players (for example see Figures 2a, b, d, e, g), and monkeys and humans displayed comparable gaze patterns during gameplay (Supplementary Figures 2g-h), suggesting convergent sampling of visual information to guide behavior.

### Human performance is correlated with well-established socio-cognitive traits

To gain insight into the cognitive and emotional processes supporting strategic competition, we explored whether PK performance is correlated with scores on several well-validated self-report psychological assays. For example, risk-taking tendencies and functional impulsivity were positively correlated with win rates for both kickers and goalies. By contrast, maximizing tendencies were negatively correlated with win rates of both kickers and goalies (Supplementary Figures 3a-c). We also found the tendency to modify self-presentation, a measure of mentalizing or theory-of-mind in daily life, was positively correlated with goalie, but not kicker, performance. This finding suggests it may be especially beneficial for goalies to “get inside the head” of their opponent to predict their actions. Similarly, emotional stability also predicted goalie but not kicker performance (Supplementary Figures 3d-e).

Regularized general linear modeling (GLM), with elastic net regression to address correlations among predictor variables, revealed that multiple socio-cognitive traits were independently correlated with PK performance. Most factors impacted kicker and goalie performance similarly and only a few factors were specific to kickers or goalies (Supplementary Figure 3f, table t1). In many cases, correlations between psychological traits and game performance were most evident in the most skilled kicker-goalie pairs (Supplementary Figures 3g-i). In addition to predicting win rates, subject-level traits also were correlated with tactical factors such as last movement timing and number of moves. For kickers, functional impulsivity was positively correlated with total number of movements, but negatively correlated with last movement timing precision. Similarly, real-life risk-taking was correlated with movement probability, but also with initiating the last movement prior to the window of advantageous timing. Kicker sensitivity to opponent behavior predicted the precision of last movement timing without impacting the total number of movements (Supplementary table t2). For goalies, functional impulsivity as well as agreeableness predicted faster response times to kicker movements (Supplementary table t3). Together, these findings indicate our virtual soccer game draws upon multiple cognitive and emotional processes that vary across individuals, thus proffering translational potential.

### Gaussian process modeling uncovers key components of strategy

**Figure 3.**
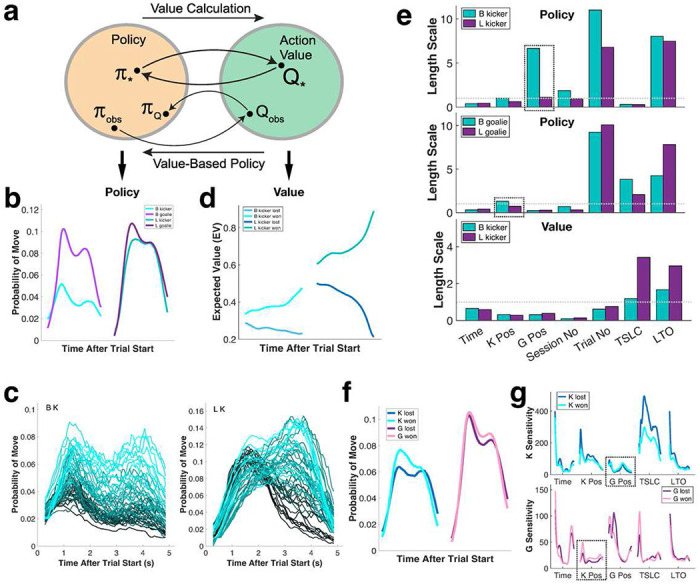
Gaussian Process (GP) models quantify movements and tactics. **a:** Relationship between policies and action values in GP models. Each policy determines an action value (rightward arrow). Conversely, a set of action values, coupled with an action selection mechanism like softmax, determines a policy (leftward arrow). **b:** Overall probabilities of movement derived from policy GP models for each monkey kicker and monkey goalie. **c:** Movement probabilities derived from policy GP models for each monkey kicker in each session. Lighter colors indicate later sessions. **d:** Overall expected values derived from value GP models, divided by actual trial outcomes. **e:** Median length scale for each model parameter in kicker policy (**top**), goalie policy (**middle**), and value (**bottom**) GP models. Length scales <1 are considered statistically significant. TSLC: time since last change/move (self). LTO: last trial outcome. **f:** Overall movement probabilities were higher in winning trials for both kickers and goalies. **g:** Sensitivities for most parameters were lower during winning trials, except for sensitivities to opponent positions (dashed boxes). X axis, time.

We adopted a computational framework known as Gaussian Process (GP) classification, previously used to quantify strategies deployed by human PK players (Iqbal et al., 2019; McDonald et al., 2019, 2020), to model the behavior of monkeys flexibly switching between playing as kicker or goalie. This framework operationally defined the decision space available to each player as a binary choice of whether to move or not at each sequential moment in the game. Each movement decision is defined as an action, and a policy function determines the probability of an action given the state of the environment (see Iqbal et al., 2019; McDonald et al., 2019 for details). To evaluate the effectiveness of each movement, we modeled each monkey’s empirical action value function, defined as the expected value of taking an action in a given state and playing according to a given policy thereafter. Each policy uniquely determines a value function for each action, while the expected value informs action selection according to policy (Figure 3a).

Our policy GP models robustly captured the overall dynamics of gameplay by monkeys (Figure 3b, Supplementary Figure 4a), and characterized movement patterns across trials and sessions (Figure 3c), echoing prior modeling of human PK performance (McDonald et al., 2019, 2020). Model comparison using held-out test data yielded a median area under the curve (AUC) of 0.66-0.73 for each policy model for each monkey player in each role (i.e., 4 policy models in total), comparable to human PK models (McDonald et al., 2019, 2020). Value GP models on the other hand yielded one set of values per kicker/goalie combination (i.e., 2 value models in total), and these models quantified the effectiveness of each tactical move by monkeys along a continuum, thus distinguishing, for example, a certain win from an uncertain win (Figure 3d).

Each GP policy and value model had 7 state variables as predictors: 1) time (i.e., ball position along the x-axis), 2) self-position (i.e., ball or bar position on the y-axis for kicker or goalie, respectively), 3) opponent position (i.e., bar or ball position on the y-axis for kicker or goalie, respectively), 4) session number, 5) trial number within a session, 6) TSLC, time since last change/move (self), and 7) LTO, last trial outcome. The dependence of the fitted GP model on each predictor was characterized by a hyperparameter, known as the length scale. Because the length scale value quantifies the change needed in a variable to alter the GP output, smaller length scales indicate more powerful predictors, with length scales <1 defined as statistically significant.

Like human players (McDonald et al., 2019, 2020), for monkeys the most powerful predictors reflected the player’s own state (i.e., self-regarding predictors: time, self-position, TSLC), rather than the opponent’s state (i.e., other-regarding predictor: other position, indicated in boxes; Figure 3e, top and middle). Kicker position was a stronger predictor for goalies, rather than vice versa, indicating a higher demand for goalies to track their opponent (Figure 3e, top and middle). Kicker movements were more strongly determined by time elapsed since their own last move (TSLC), confirming kickers’ deployment of multiple moves to regulate last movement timing (see Figures 2e–f). Finally, session number was a significant predictor for the GP policy models, indicating that, as each pair of monkeys played together for an extended period, their strategies coevolved, and their movement sequences grew more complex (see Figure 3c). For the GP value models, by contrast, time, self and other position, session number and trial number all significantly predicted expected value, whereas TSLC and LTO did not (Figure 3e, bottom).

As in McDonald et al. 2019, we also extracted a set of momentary sensitivity indices for each time point in each policy model, capturing the change in each player’s tactic in response to a small change in each state parameter. The players’ sensitivities to state changes evolved dynamically throughout gameplay (Supplementary Figure 4b). For example, for both kickers and goalies, the importance of elapsed time and LTO diminished throughout the trial (Supplementary Figure 4c). The impact of the opponent’s position, by contrast, remained uniform throughout each trial (Supplementary Figure 4c). Finally, as shown previously (see Figures 2d–e), monkeys tended to win when they moved more frequently (Figure 3f) and when they were more sensitive to their opponent’s position (Figure 3g).

### mSTS neuron firing rates signal strategic information during dynamic competition

Using 24-channel linear arrays (Microprobe Inc), we sampled a population of single mSTS neurons, many simultaneously (n = 317 neurons; maximum 18 neurons simultaneously; see Supplementary Figure 4d for recording sites), in two monkeys playing PK against each other, swapping roles every 50 trials. Despite substantial heterogeneity across neurons (Supplementary Figure 4e), individual mSTS neurons fired action potentials throughout gameplay as well as during reward delivery and responded similarly whether the monkey was playing as kicker or goalie (Supplementary Figure 4f).

**Figure 4.**
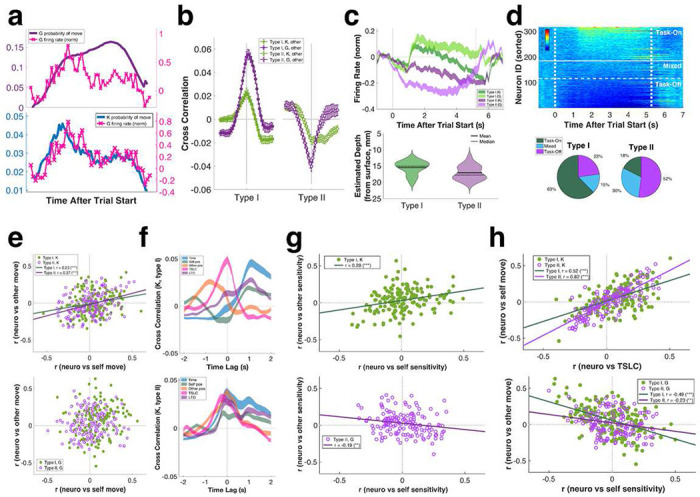
mSTS cell classification using movement probabilities from policy GP models. **a:** PSTH for an example mSTS neuron overlaid on top of goalie (i.e. self, **top**) and kicker (i.e. other, **bottom**) movement probabilities. **b:** mSTS neuronal population (n = 317) was divided into 2 types based on positive (Type I) or negative (Type II) correlations between firing rates and opponent movements. X axis, time lag (firing rate - movement probability) from −2 to 2 s. Y axis, cross correlation between firing rate and opponent movement probability. Error bars, mean ± SEM. **c: Top:** Neural activity (z-scored to pre-trial baseline) of all Type I and Type II neurons in kicker and goalie trials. Spike rates computed in 100 ms bins without smoothing. Line thickness, mean ± SEM. Vertical lines mark beginning and end of gameplay (0-5.2 s). **Bottom:** Type I neurons were recorded more dorsally to Type II neurons. **d:** Independent classification of neurons by average firing rate during gameplay (**top**) yielded similar results to model-based sorting (**bottom**). Vertical lines mark beginning and end of gameplay; horizontal lines separate task-off (i.e. suppressed, bottom), mixed (middle), and task-on (i.e., activated, top) neurons. **e:** In kickers (**top**) but not goalies (**bottom**), mSTS neurons simultaneously tracked self and other movements. **f:** Cross correlations between firing rate and model sensitivities for Type I (**top**) and Type II (**bottom**) neurons in kicker trials. X axis, time lag (firing rate - sensitivity) from −2 to 2 s. Line thickness, mean ± SEM. **g: Top:** In kickers, Type I but not Type II neurons co-represented self and opponent sensitivities. **Bottom:** In goalies, type II but not type I neurons co-represented self and opponent sensitivities, albeit in opposing directions. **h: Top:** Neurons that tracked selfmovement also encoded self-regarding sensitivities like TSLC. **Bottom:** Neurons that tracked other movement inversely signaled self-regarding sensitivities like self-position. Straight line, linear fit.

Upon closer inspection, we found individual mSTS neurons dynamically tracked either self or opponent movements during gameplay (Figure 4a). To quantify these firing rate patterns, we correlated trial-by-trial firing rates to GP policy model outputs, and then classified mSTS neurons into two groups: Type I neurons, which increased firing in response to opponent movements, and Type II neurons, which decreased firing in response to opponent movements (Type I, n = 171/317, 53.9%; Type II, n = 146/317, 46.1%) (Figure 4b). During gameplay, Type I mSTS neurons increased firing, while Type II neurons decreased firing, in comparison to pretrial baseline, regardless of whether monkeys were playing as kicker or goalie (Figure 4c, top). These two types of neurons were partially segregated anatomically as well. Type I neurons were located more superficially than were Type II neurons, potentially residing within the upper and lower banks of the superior temporal sulcus, respectively (estimated depth from cortical surface: Type I = 15.53 ± 0.16 mm, Type II = 17.10 ± 0.18 mm; Figure 4c, bottom). Type I mSTS neurons also had lower baseline firing rates compared to Type II mSTS neurons (kicker: Type I = 5.07 ± 0.38 spk/s, Type II = 7.81 ± 0.75 spk/s; goalie: Type I = 5.40 ± 0.43 spk/s, Type II = 7.94 ± 0.76 spk/s) (Supplementary Figure 4g). Thus, GP model-based cell classification corresponded well with independent sorting based on average in-task neural activity compared to pre-trial baseline (Figure 4d).

We next examined how the multitude of information previously identified as important for game performance, via behavioral as well as computational analyses, might be encoded by each type of neuron. In monkeys playing as kicker, both Type I and Type II neurons simultaneously signaled movements of both self and opponent (Figure 4e, top). This was not true for monkeys playing as goalie (Figure 4e, bottom). In other words, distinct subpopulations of mSTS neurons represented self and opponent moves in goalies. We next correlated instantaneous firing rates to GP sensitivity indices (Figure 4f), and found that, for kickers, Type I but not Type II neuron activity varied with both self and opponent sensitivity (Figure 4g, top). In goalies, by contrast, Type II but not Type I neuron activity varied with both self and opponent sensitivity, but in opposite directions (Figure 4g, bottom). Unsurprisingly, neurons that responded to self-movements also tracked self-regarding sensitivities such as TSLC (Figure 4h, top). By contrast, the firing rates of neurons that were more sensitive to opponent-movements were negatively correlated with self-regarding sensitivities (Figure 4h, bottom; also see Supplementary Figure 4h).

**Figure 5.**
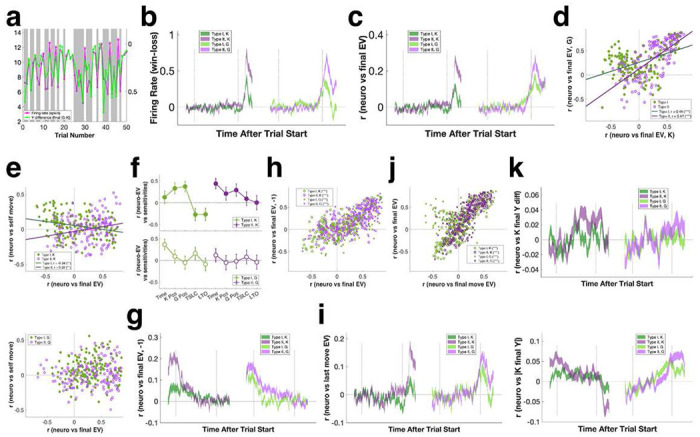
Type I and Type II neurons encode trial history. **a:** Mean firing rate per trial for example Type II neuron in a goalie, plotted over the corresponding final ball-bar Y difference. White blocks, goalie won; gray blocks, kicker won. **b:** Mean difference in firing rates between winning and losing trials for Type I and Type II neurons. Line thickness, mean ± SEM. Vertical lines mark beginning and end of gameplay. **c:** Correlations between firing rates and final EV on each trial for Type I and Type II neurons. **d:** Type I and Type II neurons signaled final EVs similarly in kickers and goalies. Straight line, linear fit. **e:** For kicker (**top)** but not goalie (**bottom**) trials, final EVs and self-move probabilities were multiplexed, but diametrically for Type I and Type II neurons. **f:** In kickers (**top)** but not goalies (**bottom**), final EVs were multiplexed with multiple self and other regarding sensitivities. **g:** Previous trial final EV influenced current trial firing rates. Line thickness, mean ± SEM. Vertical lines mark beginning and end of gameplay. **h:** Last-trial EV signals during gameplay paralleled current-trial EV during reward delivery. **i:** Correlations between firing rates and last move EV for Type I and Type II neurons. **j:** Last move EV and final EV signals were correlated. **k: Top:** Kicker but not goalie neurons encoded difference between current and last trial trajectories. **Bottom:** Goalie neurons encoded ball displacement at trial end.

Monkeys playing PK showed evidence of utilizing last-trial information to shape current trial tactics. To examine the potential neural basis of this sensitivity to history of play, we next explored whether mSTS neurons signaled task-relevant information during pre-task (−1-0 s) and post-task/peri-reward (~5-7 s) epochs. mSTS neurons tended to respond to wins and distinguished the quality of the win—that is, whether it was a sure win or not. For example, Figure 5a plots the peri-reward epoch firing rate of an example neuron recorded in a monkey playing goalie. The firing rate of this neuron was negatively correlated with final ball-bar vertical difference, thus distinguishing certain wins from uncertain wins in addition to signaling the binary outcome of the game (see Supplementary Figure 4i for another example). In other words, towards the end of the trial, both Type I and Type II neurons signaled not only game outcome (Figure 5b) but also the final expected value (EV) of the trial estimated by the value GP model (Figure 5c). These EV signals were stronger in Type II rather than Type I neurons but were otherwise similar in monkeys playing as kicker or goalie, suggesting an abstract representation of game history (Figure 5d). However, there was a subpopulation of Type I neurons with firing rates that were negatively correlated with kicker EV, but positively correlated with goalie EV (Figure 5d, upper left corner), unlike most Type I and Type II neurons (Figure 5d, upper right corner).

We found that mSTS neurons multiplexed information about final EV, self-movement (Figure 5e), and various self and other-regarding sensitivities (Figure 5f) and did so more robustly in kickers than goalies. Moreover, final EV signals were sustained into the pre-task and task phase of the following trial (Figure 5g). As a result, individual neurons signaled both last trial and current trial EV in a similar manner, independent of whether the monkey was playing as kicker or goalie (Figure 5h). These findings indicate that, despite distinct tactics deployed by kickers and goalies, mSTS neurons carried information about game outcomes and interaction history similarly independent of the specific role played by each monkey. We also considered the EV of the kicker’s final move, which was independent of the goalie’s strategy (see Figures 1c–e, cf. McDonald et al., 2019). We found mSTS neurons represented last move EV and overall trial EV in a very similar fashion (Figures 5i, j).

mSTS neurons also signaled more complex information about the dynamics of recent interactions. For example, in kickers but not in goalies mSTS neurons signaled how much the current ball trajectory deviated from the trajectory of the ball on the previous trial (Figure 5k, top), which is important for tracking trial-to-trial changes in player tactics and maintaining unpredictability for kickers. By contrast, in goalies but not in kickers, mSTS neurons signaled how much the ball was displaced from the initial starting position (Figure 5k, bottom), which may inform estimates of the strategic competence of the opponent.

### Strategic signals in mSTS during dynamic competition reflect social context

The foregoing analyses demonstrate there are two populations of neurons found at different depths within the superior temporal sulcus, which exhibit distinct tonic firing rates, display diametric task-linked modulations in activity, and dynamically signal a rich array of information relevant for supporting competitive interactions. This complex and dynamic neural representation encompasses self and opponent movements, sensitivities to self and opponent states, reward outcomes, and trial history, which together characterize patterns of strategic competition between players and distinguish player performance. Whether and to what degree these signals relate specifically to competitive *social* interactions, rather than merely reflecting sophisticated behavioral control of abstract geometric avatars on a screen, remains unanswered by these analyses.

To address this question, we examined activity of mSTS neurons in five distinct physical and social contexts (kicker: n = 77,800 trials; goalie: n = 43,700 trials): 1) *live play*, in which two monkeys competed against each other face-to-face (as described above); 2) *computer*, in which a monkey player competed against a simple computer algorithm as the opponent (the computer always moved in a straight line and never redirected); 3) *replay*, in which a monkey player competed against a recorded replay of trials previously played by another monkey (replay exhibited variability in response times and redirections but was not reactive to player moves); 4) *decoy*, in which a monkey competed against a recorded replay in the presence of another monkey, seated in the position of the opponent in the *live* condition (decoy preserved all the physical cues of live interaction, but the gameplay itself was not interactive); 5) *separate rooms*, in which two monkeys competed against each other in two separate rooms (this condition preserved all the complexity of live play without perceptual social cues such as the face, eyes, and body of the opponent monkey).

Compared with the two live competition conditions, monkey players won more and redirected less frequently in the computer, replay and decoy conditions (Supplementary Figures 5a-c), indicating sensitivity to opponent complexity and reactivity. Firing rates of mSTS neurons were more strongly modulated when monkeys competed with live opponents compared with computer, replay, or decoy opponents, even though the decoy condition shared visual, auditory, and olfactory cues with the live condition (Supplementary Figure 5d). Monkeys’ behavior in the separate rooms condition, by contrast, was indistinguishable from face-to-face live play (Supplementary Figures 5b-c, e), and mSTS neurons also responded robustly when monkeys competed in separate rooms (Supplementary Figure 5f). The same trends were evident when we only examined the subset of neurons recorded across multiple physical and social contexts (n = 250 out of 632 mSTS neurons recorded in more than one condition, and 62 recorded in at least three conditions; Supplementary Figure 5g). These findings effectively mitigate the possibility that modulations in mSTS neuronal activity during dynamic competition can be reduced to purely perceptual social representations. Instead, the signals carried by mSTS neurons are abstract and reflect the information our behavioral and computational analyses revealed as necessary for dynamic strategic competition, including tracking the behavior of the opponent, assessing their strategic tendencies, and evaluating previous interactions and outcomes.

### Inactivation of mSTS impairs strategic competition

**Figure 6.**
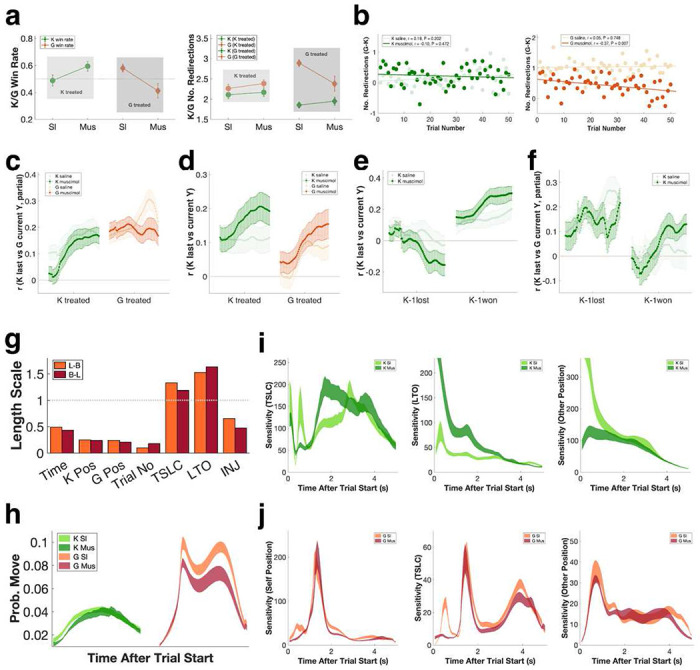
mSTS causally shapes strategic behavior. **a: Left:** Muscimol inactivation of mSTS increased win rates for kickers and decreased win rates for goalies . **Right:** Inactivation of mSTS decreased goalies’ but not kickers’ redirection rate. **b:** Muscimol progressively diminished goalies’ ability to match opponent redirections as more rounds were played. Straight line, linear fit. **c:** Muscimol negatively impacted goalies’ ability to predict kicker trajectories using trial history. **d:** mSTS inactivation increased kicker predictability. **e:** Muscimol increased use of win-stay, lose-shift strategy by kickers. **f:** Goalies were unable to take advantage of increased kicker predictability. Error bars, mean ± SEM. **g:** The median length scale for each model parameter in value GP models for L kicker-B goalie and B kicker-L goalie trials, indicating that injection type (saline or muscimol) significantly impacted trial outcome. **h:** Muscimol lowered the overall probabilities of movement for both kickers and goalies. **i:** In kickers, muscimol increased self-regarding sensitivities while lowering opponent sensitivity. **j:** In goalies, by contrast, muscimol lowered both self-regarding and opponent-regarding sensitivities.

To determine the causal contribution of mSTS neurons to dynamic strategic social competition, we injected a small amount of the GABA agonist muscimol, compared with saline control, unilaterally into mSTS and then evaluated PK game performance. We found overall win rates were significantly lower for goalies, but not for kickers, following mSTS inactivation (P = 0.007; Figure 6a, left). In fact, mSTS inactivation led to a slight improvement in kicker performance (P = 0.052; Figure 6a, left). Note that, as previously mentioned, the two monkeys always switched roles with each other in 50-trial blocks, so it was unlikely that impairment in goalie but not kicker performance simply reflected fluctuations in motivation, engagement, attention, or effort following muscimol injections. Moreover, we excluded all blocks where either player failed to complete >10% of the trials to mitigate any decrease in motivation or non-selective impairments in performance. The changes in player performance also were unlikely to be explained by spatial attentional biases such as hemi-neglect (see Supplementary Figures 6a-b).

We also observed significant muscimol-induced decreases in the overall number of redirections made by goalies (P = 0.015) but not kickers (P = 0.691; Figure 6a, right). As a result, tactically, goalies were less able to match the number of redirections made by kickers (Supplementary Figures 6c-d), and this deficit became more pronounced as the game progressed (Figure 6b, Supplementary Figure 6e). mSTS inactivation also diminished goalies’ ability to use information from prior trials to predict the kicker’s current strategy (Figure 6c). By contrast, following mSTS inactivation kickers were more likely to repeat movement trajectories (Figure 6d), shifting to a simple win-stay, lose-shift strategy (Figure 6e), although at no apparent cost to win rates (Figure 6f). Thus, inactivating mSTS impaired the selection of tactics and reduced overall strategic complexity, to the detriment of the goalie but not the kicker.

Our GP value models confirmed muscimol injection impacted game performance (Figure 6g, length scale < 1 is considered significant). The policy GP models verified that mSTS inactivation reduced the overall probability of movement, particularly for goalies (Figure 6h). Intriguingly, we found inactivating mSTS in kickers led to increased sensitivity to self-state (Figure 6i, left and middle) and decreased sensitivity to the opponent (Figure 6i, right). By contrast, inactivating mSTS in goalies reduced sensitivity to the states of both self (Figure 6j, left and middle) and opponent (Figure 6j, right), especially towards the end of the trial, when responding to kicker’s moves was most crucial for performance. Together these modeling results suggest inactivating mSTS generates specific deficits in maintaining and updating internal models of opponents and using this information to deploy effective tactics to counteract them.

## Discussion

Most prior research on social decision making has employed tasks with static, binary choices, often grounded in game theory (for example see Stephens et al., 2002; Rilling et al., 2002; Sanfey et al., 2003; Chang et al., 2012; Haroush & Williams, 2015). This approach often does not reflect the complexity of real-life interactions, which typically extend in time and dynamically evolve (Scholl et al., 2015; Camerer & Mobbs, 2017). Here we used a task that featured continuous interactions between pairs of players and demanded real-time updating of players’ models of themselves and their opponents, thus echoing recursive social interactions in real life, such as chess, poker, soccer, bargaining, and conversation (Nash, 1951; Morgenstern & Von Neumann, 1953; Camerer 1997, 2011; Chiappori et al., 2002; Moschini, 2004; Azar & Bar-Eli, 2011).

Like real life interactions, in PK the behaviors of kickers and goalies were closely entwined and delicately balanced. A skilled kicker needs to equipoise being unpredictable in their movements and precise in their timing. A skilled goalie, by contrast, needs to maintain the balance between tracking every move made by the kicker and responding swiftly to the kicker’s last move. Unlike some one-shot games (Camerer, 1997, 2011), PK performance benefits from retention of information from previous trials. Kickers tend to repeat successful tactics and eschew unsuccessful ones, but this win-stay, lose-shift tendency can be readily exploited by goalies.

We quantified individual strategic competence for both monkeys and humans, measured in several key parameters reflecting timing precision and movement unpredictability, and established a series of distributions for the general human population. Because each human player pair was unique, we used Gaussian Process (GP) classification models (McDonald et al., 2019) to evaluate the effectiveness of each tactical maneuver and disentangle the strategic competence of each player from their opponent. Both approaches confirmed monkeys played the game at least as well as unskilled human players, inviting the hypothesis that both species engage in similar cognitive and computational processes, differing quantitatively rather than qualitatively, during strategic social competition.

Current evidence for full, multi-level theory-of-mind (ToM) in nonhuman animals, including macaques, remains limited and hotly debated (Byrne & Whiten, 1991; Flombaum & Santos, 2005; Schmelz et al., 2011; Martin & Santos, 2016), in part because the behavioral tests employed are often very different from those used in humans, thus making direct comparisons difficult. In PK, we make no strong claims regarding use of ToM by either humans or monkeys. However, the close correspondence between human and monkey tactical maneuvers, overall strategies, and information sampling via eye movements, correlations between human PK performance and socio-cognitive traits including mentalizing abilities, and modulation of strategic complexity according to social context in monkeys, together invite the possibility that PK engages core component processes supporting ToM. Regardless of whether these processes are best labelled as “theory-of-mind,” our behavioral findings support use of PK as an intuitive, engaging, ecologically-valid assessment of strategic behavior and underlying cognitive processes in nonhuman primates as well as other populations with limited language or intellectual disabilities, such as small children, patients with brain damage, or individuals with autism spectrum disorders.

A prior neuroimaging study of humans playing PK reported activation of TPJ when playing against human opponents compared with playing against computers and, moreover, hemodynamic responses in TPJ were correlated with individual variation in sensitivity to opponent state (McDonald et al., 2020). We posited that if mSTS is the primate homolog of human TPJ, as suggested by resting-state fMRI studies in monkeys (Mars et al., 2013), then neurons in this area should encode information about self and opponent actions and states, and their evolution over time, just as human TPJ does (McDonald et al., 2020). Consistent with this hypothesis, we found neurons in macaque mSTS multiplex the gamut of information necessary for the selection, deployment, and evaluation of tactics, and the maintenance of strategies, including the probability of self and opponent movement, sensitivity to self and opponent states, last and current trial outcomes, and the social context.

There is a significant gap in our understanding of human TPJ with respect to its local circuitry and its relative position within the processing streams of the “social brain network,” in part due to the fact that BOLD fMRI signals often lack the resolution to identify distinct neuronal populations based on their firing rate patterns (Logothetis, 2008). Using linear array recordings, we identified two distinct populations of neurons within macaque mSTS based on their in-task activity patterns. Firing rates of Type I neurons were excited by, whereas firing rates of Type II neurons were suppressed by, opponent movements during game play. These diametric activation patterns, coupled with differing baseline firing rates and distinct depths within the STS, strongly suggest Type I and Type II neurons represent two different mSTS subregions, potentially corresponding to the upper and lower bank of the STS, respectively (Roumazeilles et al., 2021).

Successful real-world social interactions depend on flexibly and dynamically updating tactics according to the outcomes of previous interactions (Mailath & Samuelson, 2006; Camerer, 2011). Similarly, in PK information from prior trials influenced behavior of both kickers and goalies. Correspondingly, during the peri-reward epochs, both Type I and Type II neurons increased firing rates for wins, and high expected values (EVs) in general, and maintained this information deep into the next trial. mSTS neurons also signaled the precision of last moves made by kickers, paralleling hemodynamic responses in TPJ in humans playing PK (McDonald et al., 2020). Because both Type I and Type II neurons jointly signaled overall probability of winning and last move timing, we speculate both mSTS in monkeys and TPJ in humans update the expected values of tactical maneuvers based on the outcome of the most recent interaction. We hypothesize the precise control of last move timing is implemented elsewhere, possibly in dmPFC, as suggested by fMRI BOLD signals in humans playing PK (McDonald et al., 2020).

Our behavioral and computational data demonstrate kickers and goalies play PK similarly, and our electrophysiological recordings suggest the same populations of mSTS neurons are engaged during gameplay irrespective of player role. There are however several key differences in neuronal signaling in mSTS that depend on player role. Overall, Type I and Type II neurons responded similarly during the reward epoch in both kickers and goalies. During gameplay, by contrast, firing rates of Type I and Type II neurons multiplexed different types of task-relevant information when monkeys played kicker versus goalie. This difference suggests that while kickers and goalies deploy distinct tactics during gameplay, the overall maintenance of strategies and models of self and others are largely similar processes regardless of player role. In addition, several results indicate playing goalie may be more demanding than playing kicker. For example, both GP models and eye movement analysis indicate goalies pay more attention to opponent moves, and, for humans, only goalie performance is linked to validated self-report measures of the tendency to modify behavior based on the reactions of others. Moreover, mSTS neurons in goalies responded to opponent moves more strongly during gameplay and signaled the probability of winning more robustly when the outcome of competition was realized. Finally, inactivating mSTS significantly impaired PK performance in goalies but not kickers. We hypothesize these subtle but significant differences reflect the fact that, unlike kickers, who have a clear set of winning tactics–namely small, frequent moves at trial onset and one big, sudden, precisely-timed final move—goalies win by tracking the movements and strategic tendencies of the kicker and continuously adjusting their tactics in response.

Here we marshal copious behavioral and computational evidence demonstrating strong similarities between monkeys and humans in strategic competition. Moreover, our neurophysiological data, coupled with previous fMRI data in the same task (McDonald et al., 2020), strongly suggest strategic competition is mediated by similar neural mechanisms in both species. In humans, TPJ is implicated in a welter of perceptual and cognitive processes including perception of biological motion (Puce et al., 1998; Grossman et al., 2000), detection of the direction of gaze of another individual (Langton et al., 2000), memory (Wagner et al., 2005; Cabeza et al., 2008), attentional reorienting (Corbetta et al., 2008), and language (Binder et al., 2009). Integrating across these processes, many scholars contend TPJ is uniquely suited to support theory-of-mind and mentalizing in social contexts (Samson et al., 2004; Carter et al., 2012; Konovalov et al., 2021). In PK, hemodynamic responses in human TPJ track sensitivity to opponent, timing precision, and social context (McDonald et al., 2020). Our findings demonstrate neuronal activity in mSTS, which shares similar functional connectivity with human TPJ (Mars et al., 2013), signals strategic information about self and opponent, history of previous interactions, and social context, in monkeys playing PK. Inactivation of mSTS impairs the ability of monkey players, particularly goalies, to respond adaptively to opponents, demonstrating this area contributes functionally to strategic competition. Together with prior neurophysiological evidence linking mSTS to strategic cooperation (Ong et al., 2021), our findings endorse the hypothesis that multi-level intentional reasoning in humans has its origins in computations supporting strategic social interactions in nonhuman primates.

## Methods

### Human participants

Participants aged 18 to 40 years were recruited from the Philadelphia area of Pennsylvania. There were no additional selection criteria. All participants (n = 110, 49 females, 61 males) provided written informed consent, and this study was approved by the Institutional Review Board of the University of Pennsylvania.

### Non-human primates

All procedures reported in this study were approved by the Institutional Animal Care and Use Committee of the University of Pennsylvania, and designed and performed in accordance with the guide for the Public Health Service Care and Use of Laboratory Animals. Two adult male monkeys (*Macaca mulatta*; Monkey B, 18 years old, 12 kg; and Monkey L, 13 years old, 10 kg) were trained to play the competitive virtual soccer game. Monkey B was initially trained as kicker (n = 39,600 trials) and later retrained as goalie (n = 9,400 trials). Monkey L was initially trained as goalie (n = 20,400 trials) and later retrained as kicker (n = 6,400 trials). These monkeys lived in a colony with 6-10 other monkeys for the duration of the experiment, and they occupied separate cages facing the center of the room, permitting them to be in continuous visual and auditory contact outside of experimental sessions.

### Questionnaires

After signing the consent form, human participants completed a series of questionnaires and the virtual soccer game. The order of the questionnaires and the game was counterbalanced across participants.

1. Risk Propensity Scale (RPS): The RPS (Nicholson et al., 2005) is a 12-item Likert-format questionnaire assessing risk-taking in 6 domains: recreational, health, career, financial, safety, and social risks. For each of these 6 items there were two response scales, one for “now” and one for “past”. Each item was rates on a 5-point scale ranging from 1 (never) to 5 (very often). Higher scores indicate greater likelihood of risky behavior.
2. Revised Self-Monitoring Scale (RSMS): The RSMS (Lennox & Wolfe, 1984) is a 13-item Likert-format questionnaire that assesses two styles of self-monitoring behavior: Ability to Modify Self-Presentation (AMSP) and Sensitivity to the Expressive Behavior of Others (SEBO). Each item was rated on a 6-point scale ranging from 0 (strongly disagree) to 5 (strongly agree). Higher scores indicate greater likelihood of self-monitoring behavior.
3. Ten Item Personality Inventory (TIPI): The TIPI (Gosling et al., 2003) is a 10-item Likert-format questionnaire that assesses the Big Five personality dimensions: extraversion, agreeableness, conscientiousness, emotional stability, and openness to experiences. Each item consists of two descriptors, separated by a comma, using the common stem, “I see myself as:”. Each item was rated on a 7-point scale ranging from 1 (disagree strongly) to 7 (agree strongly).
4. Brief Maximizing Scale (BMS): The BMS (Nenkov et al., 2008) is a 6-item Likert-format questionnaire assessing maximizing tendency in 3 domains: alternative search, decision difficulty, and high standards. Each item was rated on a 7-point scale ranging from 1 (disagree strongly) to 7 (agree strongly). Higher scores indicate greater likelihood of maximizing behavior.
5. Dickman Impulsivity Inventory (DII): The DII (Dickman, 1990) is a 23-item questionnaire assessing 2 types of impulsivity: dysfunctional impulsivity and functional impulsivity. Each item was rated as either “agree” or “disagree”. Higher scores indicate greater likelihood of impulsive behavior.

### Behavioral paradigm

A human player sat in a chair facing a 120-Hz LCD monitor (BenQ XL2430, 24”, 1920*1080) 40 cm away, with no head restraint, and held a joystick (CTI Electronics M20U9T-Nx) placed on the table directly in front. A computer (Dell Precision Tower 5810, custom built) running MATLAB (Mathworks) and Psychtoolbox (Brainard & Vision, 1997; Pelli, 1997) was used to control all aspects of the experiment.

A monkey player sat in a primate chair (Crist Instrument) with head restrained, faced a 120-Hz LCD monitor (BenQ XL2730, 27”, 2560*1440) 60 cm away, and accessed a joystick (CTI Electronics M20U9T-Nx) mounted in a clear box attached to the front of the chair. A computer (Dell Precision Tower 5810, custom built) running MATLAB and Psychtoolbox was used to control all aspects of the experiment, including displaying visual stimuli on the monitor, sampling continuous input from the joystick (at 120 Hz), opening and closing solenoid valves (Christ Instrument) to dispense juice rewards, and sending timestamps of key behavioral events to the electrophysiology and video recording system (Plexon Inc, see below).

Three stimuli were displayed on screen—the ball (controlled by the kicker), the bar (controlled by the goalie), and the goal line—against a black background. The goal line was white. In humans, the ball was always blue and the bar was always red. In monkeys, the colors of ball and bar corresponded to the identity of the monkey player (B: blue; L: red). All stimuli on screen were luminance-balanced. The diameter of the ball was 30-40 pixels (around 1.5 visual degrees); the length of the bar was 250-350 pixels whereas the width was 20 pixels; the width of the goal line was 4 pixels and it crossed the entire screen vertically.

At the beginning of each trial, both the ball and the bar were illuminated along the horizontal midline of the screen. The ball was on the left; the bar was on the right, in front of the finish line. Throughout the trial, the ball moved across the screen (left to right) at a constant horizontal speed (~350 pixels/s). The kicker used the joystick to move the ball vertically to try to bypass the goalie, whereas the goalie used the joystick to move the bar vertically to try to block the ball. The Y-axis speeds of ball and bar were constant; the joysticks only controlled the direction of movement. A trial ended when either the kicker maneuvered the ball to the finish line, or the goalie intercepted the ball. For humans, each player’s monetary reward was linearly correlated with overall win rate (overall monetary reward ranging 12-18 dollars per subject). For monkeys, at the end of each trial the winner received a juice reward (~ 0.8 ml) while the loser received nothing. Juice was delivered via a tube with the mouth piece attached to the primate chair and controlled by a solenoid valve. In order to prevent monkeys from forming secondary associations with solenoid clicks, the valves were placed in another room. All trials were the same length (~2 s); inter-trial intervals were randomly varied (1-1.2 s). Each block of game play consisted 50 trials for humans, and 100 trials for monkeys. Each human participant played 100-300 trials in total; each monkey played 20,000-50,000 trials in total (see above).

Both human and monkey players were free to look around throughout the experiment. Human eye movements and pupil size changes were recorded with a wearable near-infrared eye tracking system, Tobii Pro Glasses 2 (Tobii), sampled at 120 Hz. Monkey eye movements and pupil size changes were recorded with an infrared eye tracking system, Eyelink 1000 Plus (SR Research, primate mount), sampled at 1,000 Hz. In addition, a video camera (Cineplex, Plexon Inc) was mounted on top of each monitor to record the facial and hand movements of the monkey facing that monitor.

During the live play condition, two humans or two monkeys played against each other using joysticks, one as kicker and the other as goalie. The two players were positioned at opposite corners of the room, allowing both visual access to their opponent. A monitor displaying the visual stimuli was positioned in front of each player (40 cm for humans, 60 cm for monkeys). Thus, each player faced a monitor directly ahead of him/her and an opponent at 45° angle (Figure 1a). All experiments were carried out in a dimly lit room (20-35 cd/m^2^) to ensure visibility of opponent. The colors, speeds, dimensions, and starting positions of the bar and the goalie bar were consistent across all sessions. The length of the goalie bar was slightly varied across sessions (ranging 250-350 pixels) to ensure an average win rate of 35-65% for both players.

During single-unit neural recordings in mSTS with linear arrays (see below), the same monkeys played against each other and switched between kicker and goalie positions in 50-trial blocks. In this setup, the two monkeys sat across from each other with one shared monitor in between, lying horizontally on a table in between the two primate chairs. The monkeys’ heads were restrained at a downward angle to ensure visibility of everything on screen. Three stimuli were displayed on screen—the ball (blue), the bar (red), and the goal line (black)—against a white background (to minimize reflection). All stimuli on screen were luminance-balanced. Player position changes were signaled with ball and bar positions on screen. More specifically, for example, when monkey B played kicker against monkey L as goalie, the ball travelled from B’s side and the goalie bar was close to L’s side. After 50 trials, the graphic presentation was flipped, and monkey L moved the ball whereas monkey B controlled the goalie bar. All trials were the same length (~5 s); inter-trial intervals were randomly varied (1-1.2 s). Each block of game play consisted 50 trials, and kicker and goalie blocks were always interleaved. One video camera stood on a tripod positioned to the side of the monitor, and another video camera was fixed on the ceiling looking down on both monkeys as well as the shared monitor.

To examine mSTS neuronal activity in different social contexts, a single monkey played against a simple computer algorithm, a recorded replay of another monkey’s previous trials, or against another live monkey sitting in a separate room. The computer algorithm moved the ball or bar in a straight line to a randomly determined final location (K vs computer, n = 15,500 trials; G vs computer, n = 16,100 trials). The recorded replay was paired either with an empty chair (a.k.a. the replay condition; K vs replay, n = 13,500 trials; G vs replay, n = 10,900 trials) or another live monkey in the same room (a.k.a. the decoy condition; K vs decoy, n = 6,600 trials). The decoy monkey could access juice reward but had no joystick to actively participate in the game. In the separate room condition, two monkeys sat in their respective rooms with doors closed, thus having no visual or auditory contact with each other (n = 4,200 trials). Note that the decoy condition was only measured in kickers, and for the separate room condition only kicker neural activity was recorded. The order in which different blocks were tested was randomized each day.

### Data analysis: behavior

Behavioral data recorded for each trial included moment-to-moment ball and bar positions, gaze, and final trial outcome. For data presentation, ball and bar positions as well as gaze were normalized to the monitor dimensions so all coordinates ranged from 0 to 1 (X = 0, Y = 0 corresponded to the upper left corner of the monitor). All subsequent data analyses were accomplished with custom MATLAB scripts. All statistical tests were two-tailed. For hypothesis testing between two samples, a non-parametric Wilcoxon signed rank test (for paired samples) or Wilcoxon rank sum test (for un-paired samples) was used. For comparison among more than two samples, an ANOVA was used together with multiple comparisons (Tukey’s HSD test) when appropriate. Correlation coefficients were estimated with Pearson’s r. Nominal factors were coded as −1s and 1s for cross correlation analyses. Nominal factors were coded as −1s and 1s and interval or ratio factors were z-scored prior to being entered into the general linear models (GLMs). GLMs with elastic net regularization was performed in MATLAB with 20-fold cross validation on regularization parameters. The results of these models were reported as estimated coefficients ± SEM with corresponding P values (without interaction items).

### Data analysis: last move timing

The advantageous time window for kicker’s last movement is predetermined by several factors: screen size (L, in pixels), ball/bar speed (v1/v2, in pixels/s), ball/bar radius (R1/R2, in pixels), and trial duration (T, in s). Thus, for each experimental setup, the position of this window remains constant, regardless of player reaction times. For example, assuming neither kicker nor goalie has made any moves prior to the last move, we have: P_kwin_ = P (0.5*L>v1*(T-rt1)>R1+R2), where the probability of kicker winning (P_kwin_) is the highest when the kicker makes the final move early enough to circumvent the goalie bar (v1*(T-rt1)>R1+R2), and late enough so that the ball does not reach the top or bottom of the screen before the trial ends (v1*(T-rt1)<0.5*L). In the human 2-player setup, L = 1080, v1 = 600, v2 = 666, T = 1.92, R1 = 15, R2 = 150. After plugging these numbers in we have 1.02<rt1<1.65. In other words, the advantageous last move window for kicker in this setup is 1.02s to 1.65s after trial start. Within the range, the probability of kicker winning depends on goalie’s RT to kicker’s last move: P_kwin_ = P (v1*(T-rt1)>v2*(T-rt1-rt2)+R1+R2). In our human dataset, median rt1 = 1.34s and median rt2 = 0.32s, which translates to an average kicker win rate of 0.44. When kicker or goalie has made multiple moves to displace the ball or bar from starting position prior to making the final shot, the probability of kicker winning can be calculated accordingly, taking into consideration the vertical position of the ball and its relative vertical distance from the goalie bar.

### Non-human primate: behavioral training

Monkeys were first trained on the virtual soccer game exclusively by playing with a computer opponent. Prior to this experiment, both monkey B and monkey L had been trained on simple tasks to gain proficiency with the joystick. The computer kicker moved the ball at constant X-axis and Y-axis speeds, and in addition the ball also traveled at an angle from the midline. In each trial, this angle was a randomly chosen integer from −40° to 40°. Similarly, the computer goalie moved the ball at a constant Y-axis speed, but in each trial the travel distance of the goalie bar was a randomly chosen integer within the full range of pixels across the Y axis on screen. Computer kicker and goalie always moved in straight lines and never redirected. Monkeys were trained with the same algorithms until their performance reached at least 80% win rate against a computer opponent. All other behavioral testing including live competition as well as all electrophysiological recordings were conducted after this training phase. The average number of training trials administrated before recording was 1,550 trials (in 8.5 days) for kickers and 3,800 trials (in 12.5 days) for goalies.

### Non-human primate: electrophysiological recording

We acquired structural magnetic resonance images (3T MRI, 1-mm slices) of each monkey’s brain to guide the placement of recording tracks and localize recording sites. We made a mask consisting of a 3mm sphere around a seed at the fundus of the right STS (X = 18.75, Y = −10.00, Z = −2.25, according to the Montreal Neurological Institute (MNI) atlas), which has a functional connectivity profile most similar to human TPJ (Mars et al., 2013). The mask was then converted to the individual monkey’s native-space structural scan to identify the target recording location using FSL’s FMRI Expert Analysis Tool (FEAT) Version 6.0.0. Detailed localizations were made using Osirix (http://www.osirix-viewer.com) or Horos (https://horosproject.org) data viewer.

All single-unit recordings were made using either single tungsten electrodes (FHC) or multichannel linear arrays (24 or 48 channels, 300 μm/channel, Microprobe Inc.). On each day of recording, a sterilized single electrode or electrode array was secured onto the recording chamber (Crist Instrument) via an X-Y stage (Crist Instrument) and an adapter (Crist Instrument). The dura was penetrated using a sterilized guide tube (22 gauge, stainless steel, custom made), and the electrode was lowered through the guide tube via a hydraulic microdrive (Kopf Instruments). Multi-unit activity and local field potentials (LFPs) were filtered and recorded using a 256-channel recording system (Plexon Inc). In addition to being guided by stereotaxic coordinates and MRI localization, each day we also confirmed recording site in STS by listening for LFP changes corresponding to gray and white matter transitions while lowering electrodes. Neurons were selected for recording based strictly on location, stability, and quality of isolation. In the first dataset, 632 mSTS neurons were recorded (357 in kickers, 275 in goalies; 345 in monkey B, 287 in monkey L), among which 36.7% were collected with electrode arrays. 39.4% of the neurons were recorded in more than one conditions, and 9.8% of the neurons were recorded in at least three conditions. In the second dataset collected exclusively with electrode arrays, 317 mSTS neurons were recorded in both kicker and goalie positions (176 in monkey B, 141 in monkey L).

### Non-human primate: electrophysiology data analysis

Activity of single neurons was isolated using a combination of principle component analysis (PCA), the Template Matching algorithm, and the Valley Seeking algorithm in three-dimensional feature space in Offline Sorter (Plexon Inc). All subsequent data analyses were accomplished with custom Neuroexplorer (Nex Technologies) and MATLAB scripts. For the first dataset, spike rates were computed during the pre-trial epoch (−0.4-0 s before trial start), the task epoch (from beginning to end of trial, 0-2.35 s), and the reward epoch (end of trial to after reward delivery, 2.35-4.5 s). For the array dataset, spike rates were computed during the pre-trial epoch (−1-0 s before trial start), the task epoch (from beginning to end of trial, 0-5 s), and the reward epoch (end of trial to after reward delivery, 5-7 s). Peri-stimulus time histograms (PSTHs) were rendered in 10 ms windows sliding in 1 ms steps with Gaussian smoothing of 5 neighboring points. For population analyses, when appropriate, we normalized firing rates to mean pre-trial firing rate, or z-scored firing rates with respect to pre-trial baseline. Using marginally different time windows and different normalization methods did not significantly change any of the results reported in this paper.

ANOVAs with multiple comparisons (Tukey’s HSD test) were used to determine the number of neurons significant for each task condition and epoch. For hypothesis testing between two samples, a non-parametric Wilcoxon signed rank test (for paired samples) or Wilcoxon rank sum test (for un-paired samples) was used. For comparison among more than two samples, an ANOVA was used together with multiple comparisons (Tukey’s HSD test) when appropriate. Correlation coefficients were estimated with Pearson’s r. Nominal factors were coded as −1s and 1s, whereas interval or ratio factors were z-scored prior to being entered into the GLMs. The results of these models were reported as estimated coefficients ± SEM with corresponding P values (without interaction terms). Elastic net regulation was done using 10-fold cross-validation and with alpha level of 0.5. All parameters within one standard error of the minimum MSE were reported as significant.

### Non-human primate: focal inactivation

GABA agonist muscimol (Sigma-Aldrich) was used to transiently and unilaterally inactivate mSTS. Muscimol was dissolved in saline to a concentration of 5 mg/ml. Saline was used as vehicle control. On each day, two injection sites (spaced approximately 2 mm apart) were selected in one animal based on prior electrophysiological recordings. At the beginning of each session the injection needle (outer diameter: 160 μm; Hamilton) was inserted through a guide tube and driven to the appropriate depth via a microdrive. 2 μl of muscimol solution or saline were infused at the site at a speed no greater than 1 μl/min. The needle was then withdrawn and the same procedure was repeated at the second site. Injections were completed in approximately 40 minutes. Behavioral measurements began immediately after the needle was retracted from the second injection site and lasted no longer than 1.5 hours. Muscimol and saline sessions were intermixed over days, and each treatment consisted of 5-7 injection sessions in each of the two animals. All injection sessions were conducted after all other behavioral and neurophysiological data were collected.

### Non-human primate: Gaussian process (GP) classification models

The details of all three types of GP models (policy, expected value, and final movement) have been described in detail elsewhere (Iqbal et al., 2019; McDonald et al., 2019). Briefly, we used GPFlow, a Gaussian process package based on the TensorFlow machine learning library, to fit separate Gaussian Process classification models to data from each monkey in each player role. Models were fit using the Sparse Variational Gaussian Process algorithm coded in GPFlow. Model hyperparameters were learned during the training run. We used a train/test split of 80/20% at the time point level to evaluate each model’s performance; test data were not used to select model parameters. As in 49, we extracted for each time point a momentary sensitivity index for each player. This index captured the change in a given player’s strategy due to small changes in game variables. For expected value and final move models, we trained GP classifiers to predict trial outcome (win/loss) as a function of instantaneous game state. For each model, dependence of the fitted GP on each input variable was characterized by a length scale hyperparameter, the value of which roughly indicated the change in a given variable needed to alter the GP output. Since our input variables were standardized to either [−1, 1] or [0, 1], a length scale ≥ 1 thus indicated that the GP meaningfully varied along the input range of a given variable.
